# Picroside II Shows Protective Functions for Severe Acute Pancreatitis in Rats by Preventing NF-*κ*B-Dependent Autophagy

**DOI:** 10.1155/2017/7085709

**Published:** 2017-06-21

**Authors:** Xuehua Piao, Baohai Liu, Lianyi Guo, Fanji Meng, Leming Gao

**Affiliations:** ^1^Department of Traditional Chinese Medicine, The First Affiliated Hospital, Jinzhou Medical University, Jinzhou 121001, China; ^2^Department of Gastroenterology, The First Affiliated Hospital, Jinzhou Medical University, Jinzhou 121001, China; ^3^Department of Cardiology, The Fourth Affiliated Hospital, Harbin Medical University, Harbin 150001, China; ^4^Peking University of Stomatology 2nd Dental Center, Jinzhou 121004, China

## Abstract

Picroside II, from the herb *Picrorhiza scrophulariiflora Pennell*, has antioxidant and anti-inflammatory activities. However, its function on severe acute pancreatitis (SAP) and molecular mechanism remains unknown. The effects of picroside II on the SAP induced by cerulean were investigated. SAP rats were treated with picroside II (25 mg/kg). The severity of SAP was evaluated by using biochemical and histological analyses. Pancreatic cancer cell PANC-1 was transfected with ptfLC3 (an indicator of autophagic activity), pcDNA3.1-NF-*κ*B (nuclear factor kappa B), and pTZU6+1-NF-*κ*B-shRNA and then treated with picroside II. Relative molecules related with NF-*κ*B-dependent autophagy were detected by using Western blot. Autophagic activities were observed by phase-contrast and fluorescent microscopes. Acetylated LC3 was detected by immunoprecipitation. The results showed that picroside II treatment reduced the level of ALT, AST, NF-*κ*B, IL-1*β*, IL-6, TNF-*α*, and SIRT1 (NAD^+^-dependent deacetylase) and increased the level of SOD and GSH. The autophagic activity was reduced when NF-*κ*B was silenced, and the levels of TNF-*α* and SIRT1 were reduced. In contrast, the overexpression of NF-*κ*B increased autophagic activity and the level of TNF-*α*, which activated SIRT1. SIRT1 deacetylated LC3 and increased autophagic activities. Picroside II ameliorates SAP by improving antioxidant and anti-inflammtory activities of SAP models via NF-*κ*B-dependent autophagy.

## 1. Introduction

Severe acute pancreatitis (SAP) is a disease with high morbidity and mortality. It can induce vascular leakage, shock, systemic inflammatory response syndrome, and even organ dysfunctions [1]. Medical therapy is still the main option for treating SAP. However, the medicine has side effects and limit its usage in the therapy of SAP [2]. It is necessary to explore new treatment for SAP therapy.

**Figure 1:**
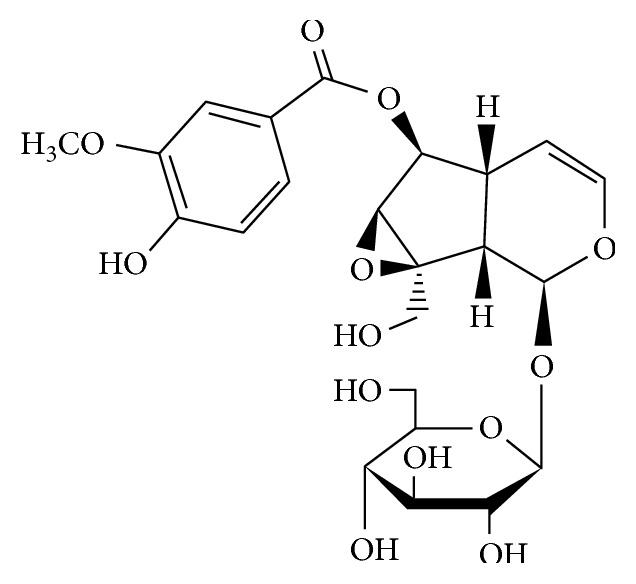
Chemical structure of picroside II.

*Picrorhiza scrophulariiflora Pennell* belongs to figwort family of flowering plants, and its active medicinal constituents are obtained from its dried root and rhizomes. It has been traditionally used to treat disorders of the liver, upper respiratory tract diseases, dyspepsia, chronic diarrhea, and scorpion sting [3]. Picroside II (h-D-glucopyranoside,1a,1b,2,5a,6,6a-hexahydro-6-[(4-hydroxy-3-methoxybenzoyl)oxy]-1a(hydroxymethyl)oxirenocyclopenta[1,2-c]pyran-2-yl) is one of the most effective components extracted from *Picrorhiza scrophulariiflora* (Figure 1) [4]. In previous experiments, we confirmed that picroside II protected cardiomyocytes through reduction of ROS production, enhancement of the activity of antioxidant defense, and anti-apoptotic effects [5, 6]. Also, many previous studies have shown that picroside II has a wide range of pharmacological effects, including neuroprotective [7], hepatoprotective [8], antioxidation [9], and anti-inflammatory activities [10]. Picroside II may potentially show beneficial effects on SAP as NF-*κ*B inhibitors with few side effects [11]. However, the exact molecular mechanism remains unclear.

The changes of cytokines have been determined in the development of SAP. The inflammatory pathways participate in this disease from the early stage to the end. Local inflammatory reaction in pancreas leads to systemic inflammatory response syndrome (SIRS) and multiorgan failure (MOF), which is believed to be the main cause of mortality [12]. Inflammatory cytokines include interleukin 1 (IL-1) *β* [13], IL-6 [14], and tumor necrosis factor- (TNF-) *α* [15] are considered to be required for SAP development. A recent study has shown that proinflammatory cytokines such as IL-6 and TNF-*α* play a central role in the initiation and progression of SAP [16]. TNF-*α* is thought to induce a cascade of other inflammatory cytokines and activate various immune cells, thus inducing the proinflammatory response [17, 18]. It has been confirmed that serum IL-6 level is a very good discriminator of SAP and can be used as an early marker of SAP [3]. TNF-*α* and IL-6 aggravate SAP and increase plasma extravasation and induce leukocyte adherence, result in SIRS (the systemic inflammatory response syndrome) and MODS (the multiple organ dysfunction syndrome) [19]. Preventing the activity of the cytokines may attenuate the systemic complications of SAP.

NF-*κ*B pathway plays an important role in inflammatory activities by affecting proinflammatory cytokines [20]. Blocking the activation of NF-*κ*B appears to reduce the inflammatory response in SAP [21]. TNF-*α* is a multifunctional proinflammatory cytokine and is significantly correlated with the expression of NF-*κ*B [22]. On the other hand, overexpression of NF-*κ*B promotes the expression of TNF-alpha-induced-SIRT1. NF-*κ*B silence will lead to a reduction in the expression of TNF-alpha-induced-SIRT1 [23]. SIRT1, as NAD^+^-dependent deacetylase, can regulate the acetylated situation of many important immune genes [24–26]. Enhanced level of SIRT1 will reduce the acetylation of endogenous microtubule-associated protein 1A/1B light chain 3 (LC3) and lead to autophagic activities [27]. Autophagy action is closely correlated with apoptosis activities [28], which will aggregate the severity of SAP [29].

Picroside II treatment may be associated with changes of these molecules and affect the autophagy and anti-inflammatory activities. This study was designed to determine the probable molecular mechanisms of picroside II in ameliorating cerulein-induced SAP in rats [30].

## 2. Materials and Methods

### 2.1. Animals

48 male Sprague-Dawley (SD) rats weighing 250–300 g were obtained from the Experiment Animal Center, Jinzhou Medical University (Jinzhou, China). The rats were allowed to adapt to our laboratory environment for one week before the experiment. They were housed in plastic cages containing corn chip bedding and were maintained on a 12 h light 12 h dark cycle (07 : 00–19 : 00 h, light cycle; 19 : 00–07 : 00 h, dark cycle) with a room temperature of 22 ± 1°C and a humidity of 65–70%. Water and food were available ad libitum. This animal study was approved by the Ethical and Research Committee of Jinzhou Medical University (2016CN028).

### 2.2. Drugs

Cerulein was purchased from Sigma-Aldrich (St. Louis, MO, USA). Picroside II (purity > 99%) was purchased from Best Regent Limited Company (Chengdu, China, batch number: MUST-12031501), dissolved in dimethyl-sulphoxide (DMSO), and diluted in 0.9% saline solution. The total amount of DMSO was less than 1%, which was considered of no significance in the assays. Cerulein was dissolved in normal saline (0.9% NaCl) immediately before use.

### 2.3. Induction of Severe Acute Pancreatitis and Administration of Picroside II

Severe acute pancreatitis (SAP) was induced in rats by intraperitoneal injections of cerulein at intervals of 1 h for five times [31]. The control rats were given saline (0.9% NaCl) solution instead of cerulein. After a two-hour cerulein injection, fifteen SAP rats were administrated with different concentration picroside II (12.5, 25, and 50 mg/kg) in 250 *μ*L 0.9% saline solution via tail vein, while those in the control group were simultaneously injected 250 *μ*L 0.9% saline solution. After 3 hours, the rats were anesthetized with pentobarbital (40 mg/kg i.p.), blood samples were drawn, and the animals were killed. The pancreas was quickly removed and frozen at −80°C until use. The optimal dose of picroside II was evaluated based on the levels of serum amylase and lipase, biochemical indicators, histological scores, and pancreatic damage as introduced below.

After the selection of optimal dose of picroside II, 45 rats were randomly divided into three groups (healthy control, 25 mg/kg saline solution; SAP model, 25 mg/kg saline solution; and picroside II-treated SAP model, 25 mg/kg picroside II) and each group was further divided into three subgroups with 5 rats, respectively. All rats were fasted overnight with continued access to water. Rats were sacrificed under anesthesia at three time points (3, 6, and 12 h) after the administration of picroside II. The pancreas was rapidly removed from each rat, and a portion of pancreas was fixed in 4% paraformaldehyde for overnight at 4°C and embedded in paraffin wax or frozen immediately at −80°C. The remaining pancreas was quickly put into liquid nitrogen and frozen at −80°C until further use. Blood samples were maintained at room temperature for 2 h before centrifugation (~3000 ×g) at 4°C for 15 min, and serum was stored at −80°C.

### 2.4. Biochemical Analysis

Serum levels of amylase [32, 33] and lipase [34] are two important biomarkers of SAP. The serum levels of two enzymes were measured after SAP establishment. Serums amylase and lipase were detected by using amylase assay kit from Pharmacia & Upjohn (Kalamazoo, MI, USA) and pancreatic lipase assay kit from Biocompare (South San Francisco, CA, USA). The serum activity of superoxide dismutase (SOD) was measured by formazan—WST method [35]. The serum concentration of reduced glutathione (GSH) was determined by the method of dithiobis-2-nitrobenzoic acid (DTNB) [36]. The serum concentrations of aspartate aminotransaminase (AST) and alanine aminotransferase (ALT) were evaluated by using Hitachi 7170A/7180 Biochemical Analyzer (Hitachi, Japan).

### 2.5. ELISA Test

Blood was collected after decapitation and incubated overnight at 4°C. Serum was recovered after centrifugation and aliquot. The aliquots were stored at −20°C for later tests. The serum level of NF-*κ*B was measured by using a rat NF-*κ*B ELISA kit (CUSABIO and CusAb, College Park, MD, USA). The serum levels of IL-1*β*, IL-6, and TNF-*α* were measured by using the kits from Wuhan Boster Biological Engineering Co. Ltd. (Wuhan, China). Serum level of SIRT1 was measured by using rat SIRT1 ELISA kit from Shanghai Sunred Biotechnology Co. Ltd (Shanghai, China).

### 2.6. Histopathologic Analysis

The removed entire pancreatic tissues were immersion fixed in 4% paraformaldehyde for 24 h, followed by dehydrating and embedding in paraffin using a routine protocol. The paraffin-embedded tissue samples were cut at 4 mm thick at longitudinal section and stained with hematoxylin and eosin (H&E). The slides were scored by two blinded experienced pathologists, and the histopathological changes of the pancreatic tissue were evaluated by light microscopy. Two slides and ten fields were examined for histopathological analysis in each pancreas. The histopathology scoring criteria were edema, acinar cell necrosis, hemorrhages, and inflammation. The scoring system was used for histopathological evaluation, as shown in Table 1, and the final score of each section was the summation of each pathological parameter.

### 2.7. Immunohistochemistry

Before immunohistochemistry analysis, all slides were air-dried. The sections were fixed in four percent of paraformaldehyde for 10 min, washed, and permeabilised in PBS buffer for 20 min. The sections were blocked in 10% horse serum and 5% BSA for one hour. Anti-NF-*κ*B antibody (Catalogue number ab36104, Abcam, Cambridge, MA, USA) was applied to the sections for 1 h at room temperature. The stain was performed by using hematoxylin stain as background color and 3,3-diaminobenzidine (DAB) stain to reveal positively stained tissue areas according to an earlier report [37].

### 2.8. pcDNA3.1-NF-*κ*B Reconstruction

NF-*κ*B gene was amplified by using the primers (sense primer, 5′-GTGAGCTAGCatggagagttgctacaaccc-3′; antisense primer, 5′-CTGAGAATTCcgccaggccgaacaggcgcg-3′). The PCR production was linked to pcDNA3.1 vector at *Nhe*I and *EcoR*I sites, and the vector pcDNA3.1-NF-*κ*B was reconstructed. The vectors were amplified in *E. coli* and isolated by using a Plasmid Miniprep Kit (Clontech, Palo Alto, CA, USA). The sequences were confirmed via DNA sequencing.

### 2.9. Constructs for NF-*κ*B shRNA

NF-*κ*B coding sequence and the reverse complementary sequence were synthesized: siNF-*κ*B, sense 5′-TCGACcccactgtcaagatctgtaactTTGGagttacagatcttgacagtggg TTTTT-3′; antisense 5′-CTAGAAAAAcccactgtcaagatctgtaactCCAAagttacagatcttgacagtgggGG-3′. *Sal*I and *Xba*I restriction sites were added to the either end of the oligos and linked to pTZU6+ 1, and thus recombinant plasmid pTZU6+1-NF-*κ*B-shRNA was constructed.

### 2.10. Cell Culture and Plasmid Coinfection

Human pancreatic cell lines PANC-1 and pancreatic *β*-cell lines INS-1 were purchased from The Cell Bank of Type Culture Collection of Chinese Academy of Sciences (Shanghai, China). Islets were isolated from healthy rats and model rats and dispersed into single cells according to an earlier report [38]. PANC-1 cells were cultured in DMEM, and INS-1 and isolated islet cells were cultured in RPMI 1640 with 10% (*v/v*) FCS, 10 mM HEPES and 2 mM glutamine, 100 *μ*g/mL penicillin, and 100 *μ*g/mL streptomycin at 37°C and 5% CO_2_. Pancreatic cancer cell line PANC-1 was transfected with the plasmids ptfLC3 (an indicator of autophagic activity, Addgene; ID 21074, Cambridge, MA, USA), pcDNA3.1-NF-*κ*B, and pTZU6+1-shRNA-NF-*κ*B. Transfection was performed via Lipofectamine 2000TM (Invitrogen, Carlsbad, CA, USA). After a two-day transfection, the antibiotic-resistant cells were collected by using 200 *μ*g/mL G-418. The strain was cultured at the same condition for three days. 25 mg/L picroside II was added to PANC-1, INS-1, and isolated islet cells and cultured in DMEM at 37°C and 5% CO_2_.

### 2.11. Western Blot Analysis

Total proteins were extracted by mammalian cell extraction kit (Wuhan Boster Biological Engineering Co. Ltd., China). Extracts were fractionated by using SDS-PAGE. The proteins were then electrotransferred to PVDF membrane (Millipore, MA, USA). The membrane was soaked with 5% skim milk in TBST buffer and then incubated with the following first antibodies at room temperature for one hour: anti-NF-*κ*B antibody, SIRT1 antibody (Catalogue number sc-74465, Santa Cruz, CA, USA), anti-TNF-*α* antibody (Catalogue number ab6671, Abcam), anti-LC3B antibody (Catalogue number ab63817, Abcam), anti-acetyl lysine antibody (Catalogue number ab80178, Abcam), and or anti-GAPDH antibody (Catalogue number ab9485, Abcam). After washing, they were interacted with the horseradish peroxidase- (HRP-) conjugated secondary antibody (Catalogue number ab6721, Abcam) for one hour. The membranes were visualized using enhanced ECL (Millipore, Billerica, MA, USA) and a ChemiDoc MP imaging system (Bio-Rad, Hercules, Ca, USA).

### 2.12. Coimmunoprecipitation Analysis

Human pancreatic cell lines PANC-1 lysates were incubated with anti-acetylated-Lys, anti-LC3, anti-SIRT1, anti-IgG antibodies, and PureProteome Protein A/G Mix Magnetic Beads. The proteins were separated by SDS-PAGE and transferred to a PVDF membrane as abovementioned. The acetylation LC3 was determined via Quantity One software.

### 2.13. Data Analysis

Data were expressed as means ± SD. Statistical analysis was performed with SPSS 17.0 statistical software. The Student *t*-test or analysis of variance was used for data analysis. A value of *P* < 0.05 was considered statistically significant.

## 3. Results

### 3.1. Establishment of SAP Model

**Figure 2:**
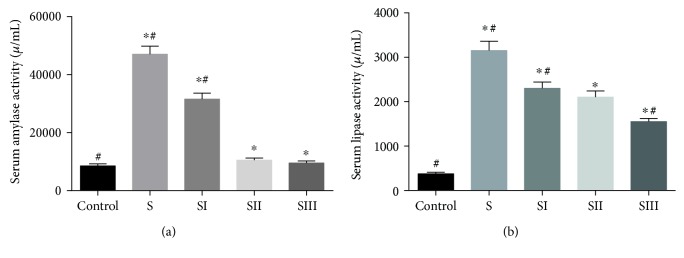
The effects of picroside II on the serum amylase and lipase activities. (a) The effects of picroside II on the serum amylase activities. (b) The effects of picroside II on the serum lipase activities. S, SAP models. SI, SAP models were treated with 12.5 mg/kg picroside II. SII, SAP models were treated with 25 mg/kg picroside II. SIII, SAP models were treated with 50 mg/kg picroside II. Data were presented as means ± SD from five independent experiments. ^∗^*P* < 0.05 versus the control group; ^#^*P* < 0.05 versus the picroside II-treated group (25 mg/kg).

The results demonstrated that the activities of serum amylase (Figure 2(a)) and lipase (Figure 2(b)) were higher in the rats treated with cerulean than the controls (*P* < 0.05). Picroside II treatment reduced the levels of serum amylase and lipase in the rats treated with cerulean (*P* < 0.05), and there was no significant difference between 25 mg/kg and 50 mg/kg, so final 25 mg/kg might be an optional dosage for the subsequent experiments.

According to histological injury scores, the score severity of the pancreatic injury could be reflected by the scores of edema, acinar cell necrosis, hemorrhage, inflammation, and perivascular infiltration. Histopathological scores of different groups were summarized in Table 2. There was a significant difference for the degrees of pancreatic edema, acinar cell necrosis, hemorrhage, inflammation, and perivascular infiltration between the control and SAP groups (Table 2). The SAP model was established according to an earlier report [39]. Picroside II treatment reduced these score, and there was a significant difference between 12.5 and 25 mg/kg picroside II treatment, but there was no significant difference between 25 and 50 mg/kg picroside II treatment. Twenty-five mg/kg picroside II was an optimal dosage for SAP therapy.

### 3.2. Picroside II Treatment Increase Antioxidant Activities in Rats

Serum biochemical index analysis showed that serums ALT and AST reached the highest level in SAP models when compared with other groups (Table 3) (*P* < 0.05). In contrast, the serums SOD and GSH reached the lowest level. Picroside II treatment reduced serum levels of ALT and AST and increased the levels of SOD and GSH in SAP models. Serums ALT and AST were at the lowest level, and SOD and GSH were at the highest level in CG (Table 3) (*P* < 0.05).

### 3.3. Picroside II Treatment Reduced Serum Levels of NF-*κ*B in Pancreas Tissues of Rats

**Figure 3:**
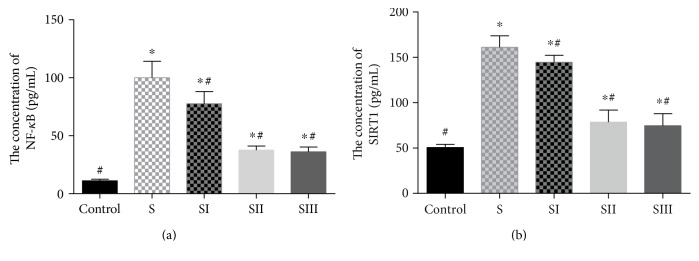
The effects of picroside II on the serum level of NF-*κ*B. Data were presented as means ± SD from five independent experiments. ^∗^*P* < 0.05 versus the control group; ^#^*P* < 0.05 versus the picroside II-treated group (25 mg/kg).

The results demonstrated that the levels of serum NF-*κ*B (Figure 3(a)) were higher in the rats treated with cerulean than in the controls (*P* < 0.05). Picroside II treatment reduced the levels of serum NF-*κ*B in the rats treated with cerulean (*P* < 0.05), and there was no significant difference for serum level of NF-*κ*B between 25 mg/kg and 50 mg/kg (*P* < 0.05). In the similar case, the levels of serum SIRT1 (Figure 3(b)) were higher in the rats treated with cerulean than in the controls (*P* < 0.05). Picroside II treatment reduced the levels of serum SIRT1 in the rats treated with cerulean (*P* < 0.05), and there was no significant difference for serum level of SIRT1 between 25 mg/kg and 50 mg/kg (*P* < 0.05).

### 3.4. Picroside II Reduced Serum Levels of Inflammatory Cytokines

**Figure 4:**
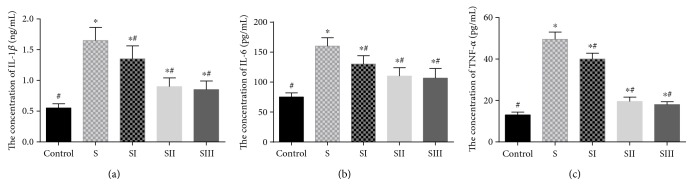
The effects of picroside II on the serum levels of inflammatory cytokines. (a) The effects of picroside II on the serum levels of IL-1*β*. (b) The effects of picroside II on the serum levels of IL-6. (c) The effects of picroside II on the serum levels of TNF-*α*. Data were presented as means ± SD from five independent experiments. ^∗^*P* < 0.05 versus the control group; ^#^*P* < 0.05 versus the picroside II-treated group (25 mg/kg).

The levels of serum IL-1*β* (Figure 4(a)), IL-6 (Figure 4(b)), and TNF-*α* (Figure 4(c)) were higher in the rats treated with cerulean than in the controls (*P* < 0.05). Picroside II treatment reduced the levels of serum inflammatory cytokines in the rats treated with cerulean (*P* < 0.05), and there was no significant difference for the serum levels of inflammatory cytokines between 25 mg/kg and 50 mg/kg (*P* < 0.05).

### 3.5. Picroside II Treatment Reduced Histological Injury Scores

**Figure 5:**
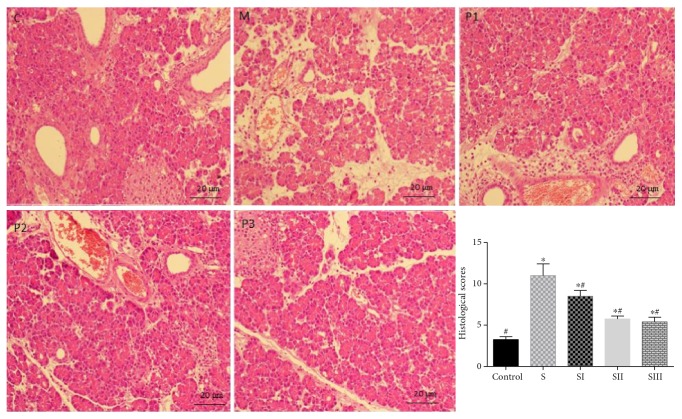
Hematoxylin and eosin (H&E) stain of pancreas tissues of rats. C, controls. M, SAP models. P1, SAP models were treated with 12.5 mg/kg picroside II. PII, SAP models were treated with 25 mg/kg picroside II. PIII, SAP models were treated with 50 mg/kg picroside II. Data were presented as means ± SD from five independent experiments. ^∗^*P* < 0.05 versus the control group; ^#^*P* < 0.05 versus the picroside II-treated group (25 mg/kg).

H&E stain showed that the histological injury scores were higher in the rats treated with cerulean than in the controls (*P* < 0.05, Figure 5). Picroside II treatment reduced the histological injury scores in the rats treated with cerulean (*P* < 0.05), and there was no significant difference for histological injury scores between 25 mg/kg and 50 mg/kg (*P* < 0.05). Therefore, all the above results showed that 25 mg/kg was chosen as the final dosage for the subsequent experiments.

### 3.6. The Effects of Treating Time on Histological Injury Scores

**Figure 6:**
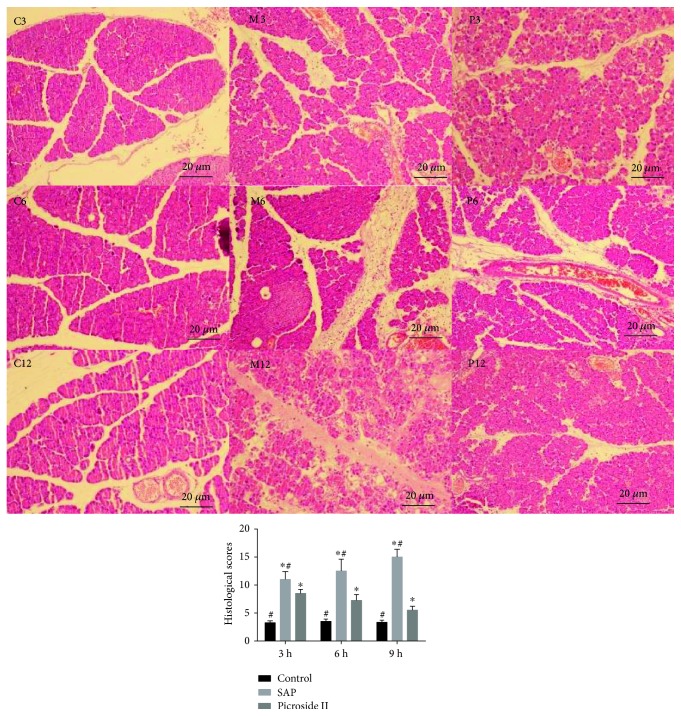
Hematoxylin and eosin (H&E) stain analysis for the effects of treat time on pancreas tissues of rats. C3, C6, and C12, the healthy rats were treated with 25 mg/kg 0.9% saline solution for 3, 6, and 12 h, respectively. M3, M6, and M12, the SAP models were treated with 25 mg/kg 0.9% saline solution for 3, 6, and 12 h, respectively. P3, P6, and P12, SAP models were treated with 25 mg/kg picroside II for 3, 6, and 12 h, respectively. Data were presented as means ± SD from five independent experiments. ^∗^*P* < 0.05 versus the control group; ^#^*P* < 0.05 versus the picroside II-treated group (25 mg/kg).

Histological injury scores were measured after 3 h, 6 h, and 9 h saline solution treatment or picroside II treatment. H&E stain showed that the histological injury scores were higher in the rats treated with cerulean with the time increasing and reached the highest level after 12 h (*P* < 0.05, Figure 6). Picroside II treatment reduced the histological injury scores in the rats treated with cerulean with the time increasing and reached the lowest level after 12 h (*P* < 0.05). There was no significant difference for histological injury scores in the controls (*P* > 0.05).

### 3.7. The Effects of Treating Time on the Expression of NF-*κ*B

**Figure 7:**
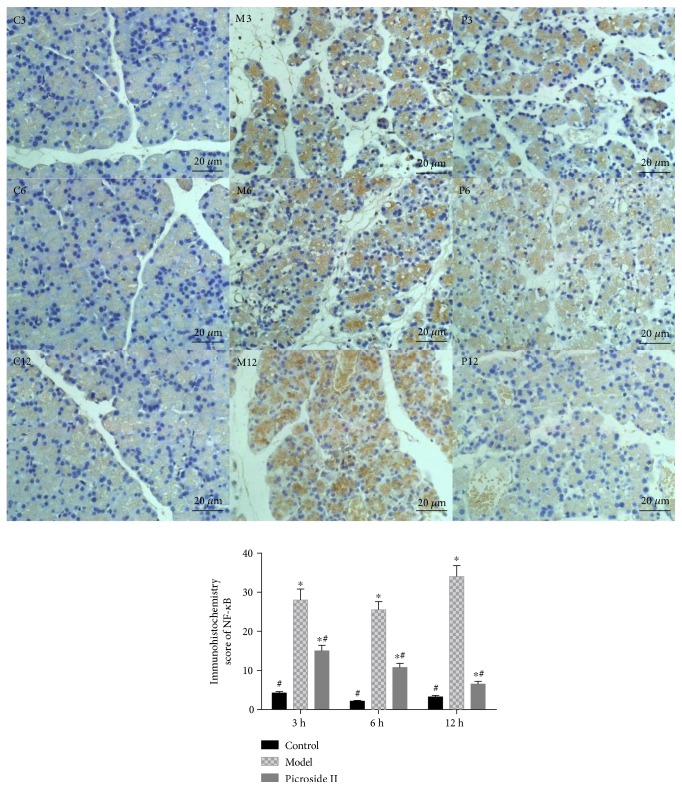
Immunohistochemical analysis of NF-*κ*B expression in pancreas tissues of rats. C3, C6, and C12, the healthy rats were treated with 25 mg/kg 0.9% saline solution for 3, 6, and 12 h, respectively. M3, M6, and M12, the SAP model were treated with 25 mg/kg 0.9% saline solution for 3, 6, and 12 h, respectively. P3, P6, and P12, the SAP models were treated with 25 mg/kg picroside II for 3, 6, and 12 h, respectively. Data were presented as means ± SD from five independent experiments. ^∗^*P* < 0.05 versus the control group; ^#^*P* < 0.05 versus the picroside II-treated group (25 mg/kg).

Histochemistry analysis showed that the protein level of NF-*κ*B was higher in the rats treated with cerulean with the time increasing and reached the highest level after 12 h (*P* < 0.05, Figure 7). Picroside II treatment reduced the expression of NF-*κ*B in the rats treated with cerulean with the time increasing and reached the lowest level after 12 h (*P* < 0.05). There was no significant difference for the protein level of NF-*κ*B in the controls from 3 h to 9 h test (*P* > 0.05).

### 3.8. Picroside II Treatment Reduces Autophagy

**Figure 8:**
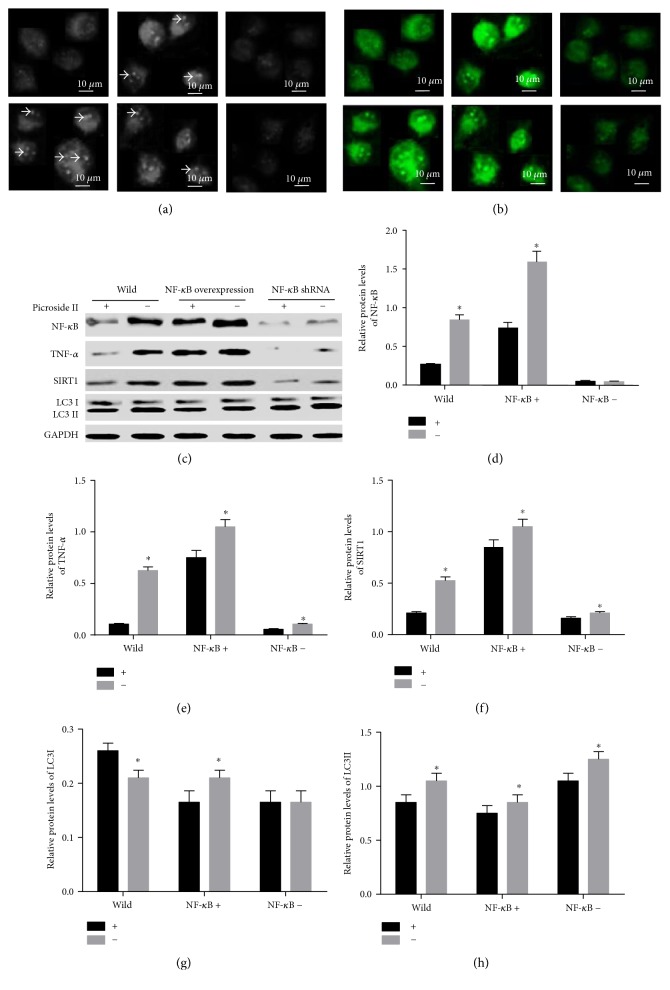
Picroside II reduced the level of SIRT1 and LC3-induced autophagy. (a) The arrows show the autophagic vacuolization in pancreas cells confected with NF-*κ*B or NF-*κ*B shRNA under a phase-contrast microscope (×200). (b) Distribution of GFP-LC3 in pancreas cells confected with NF-*κ*B or NF-*κ*B shRNA under a fluorescent microscope (×200). (c) Western blot analysis of the expression of NF-*κ*B, TNF-*α*, SIRT1, and LC3. (d) Relative protein level of NF-*κ*B in different groups. (e) Relative protein level of TNF-*α* in different groups. (f) Relative protein level of SIRT1 in different groups. (g) Relative protein level of LC3 I in different groups. (h) Relative protein level of LC3 II in different groups. NF-*κ*B+, NF-*κ*B overexpression. NF-*κ*B−, NF-*κ*B shRNA. +, picroside II treatment. −, without picroside II treatment. ^∗^*P* < 0.05 via a picroside II-treated group.

The results showed that picroside II treatment reduced autophagy vacuoles in pancreatic cells when comparing the cells without picroside II treatment (Figure 8(a), arrow showed). On the other hand, green fluorescence was enhanced with the expression of GFP-LC3 (Figure 8(b)). However, the autophagic activities could not be affected by picroside II treatment when NF-*κ*B was overexpressed or silenced. The autophagy vacuoles were increased (Figure 8(a)), and green fluorescence was enhanced with the expression of GFP-LC3 (Figure 8(b)) when NF-*κ*B was overexpressed. The levels of TNF-*α* and SIRT1 were increased (Figure 8(c)). In contrast, the number of autophagy vacuoles was reduced (Figure 8(a)) and green fluorescence was reduced with the expression of GFP-LC3 (Figure 8(b)) when NF-*κ*B was silenced. The levels of TNF-*α* and SIRT1 were increased (Figure 8(c)). The results suggest that picroside II may reduce autophagy by reducing the levels of LC3 and SIRT1 (Figure 8(c)). Picroside II reduced the expression of NF-*κ*B (Figure 8(d)), TNF-*α* (Figure 8(e)), SIRT1 (Figure 8(f)), and LC3 II (Figure 8(h)) in the wild cells PANC-1, the cells with the overexpression of NF-*κ*B and NF-*κ*B shRNA.

### 3.9. The Effects of Picroside II on the Expression of NF-*κ*B, TNF-*α*, SIRT1, and LC3 in Different Cells

**Figure 9:**
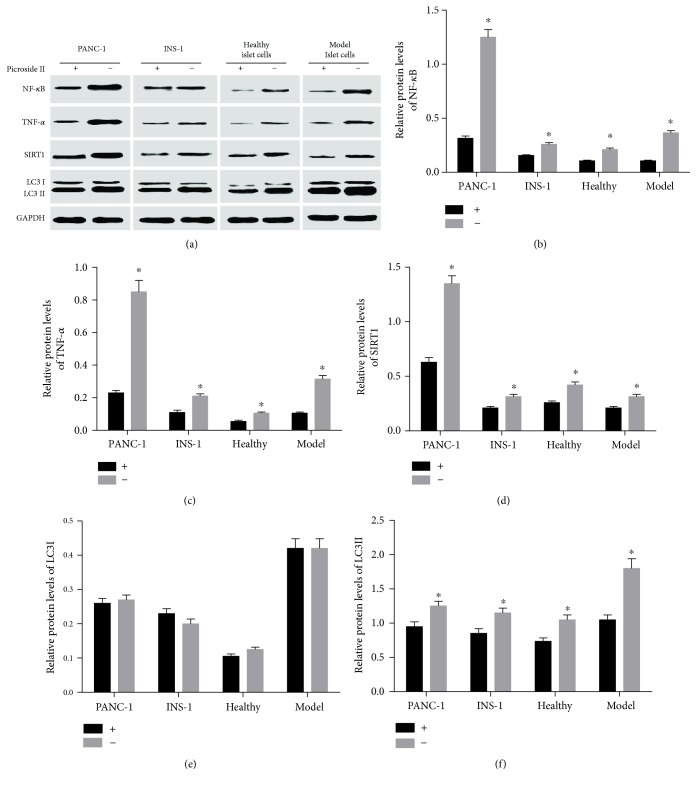
The effects of picroside II on the expression of NF-*κ*B, TNF-*α*, SIRT1, and LC3 in different cells. (a) Western blot analysis of the expression of NF-*κ*B, TNF-*α*, SIRT1, and LC3 expression. (b) Relative protein level of NF-*κ*B in different groups. (c) Relative protein level of TNF-*α* in different groups. (d) Relative protein level of SIRT1 in different groups. (e) Relative protein level of LC3 I in different groups. (f) Relative protein level of LC3 II in different groups. +, picroside II treatment. −, without picroside II treatment. Healthy, islet cells from healthy rats. Model, islet cells from model rats. ^∗^*P* < 0.05 via the picroside II-treated group.

Western blot analysis showed that picroside II treatment had the similar effects on the expression of NF-*κ*B, TNF-*α*, SIRT1, and LC3 in these cells (Figure 9(a)). Picroside II reduced the expression of NF-*κ*B, TNF-*α*, SIRT1, and LC3 II in the cells PANC-1 (Figure 9(b)), INS-1(Figure 9(c)), islet cells from healthy (Figure 9(d)), and model rats (Figure 9(e)) (*P* < 0.05). The results suggest that pancreatic cancer cells are similar with normal pancreatic cells with the same changing trend for the expression of NF-*κ*B, TNF-*α*, SIRT1, and LC3 II after picroside II treatment.

### 3.10. Picroside II Increases Acetylation of LC3 by Increasing SIRT1

**Figure 10:**
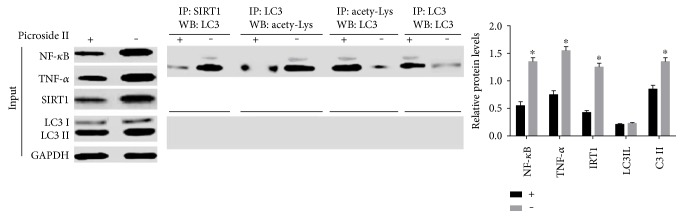
Picroside II reduced the deacetylation of endogenous LC3 by upregulating the level of SIRT1. Picroside II reduced the level of TNF-*α* by reducing the level NF-*κ*B, which deacetylated LC3. The acetylated LC3 was detected by using an IP test and Western blot. +, picroside II treatment. −, without picroside II treatment. ^∗^*P* < 0.05 via the picroside II-treated group.

Coimmunoprecipitation (co-IP) was carried out to measure the binding of SIRT1 and LC3. The results demonstrated that picroside II reduced the binding between SIRT1 and LC3, which reduced deacetylated LC3 (Figure 10). These results demonstrated that picroside II reduced the acetylation of LC3 by increasing SIRT1.

## 4. Discussion

Picroside II is known to have various beneficial effects on human health, including neuroprotective [40], hepatoprotective [41], antioxidation [42], and anti-inflammatory effects [11]. This study showed that picroside II ameliorated the severity of cerulein-induced SAP rats. The effects of picroside II on cerulein-induced SAP rats were assessed based on the improvement of edema, acinar cell necrosis, hemorrhages, and inflammation by preservation of the pancreatic architecture in comparison with the rats from the SAP group without picroside II treatment. Our findings demonstrated that picroside II treatment significantly suppressed the serum activities of amylase and lipase (Figure 2).

Many factors are involved in the process of the pathogenesis of SAP, and the accurate mechanisms are still unclear. Inflammation mediators, such as the proinflammatory cytokines, IL-1*β* [43], IL-6 [14], and TNF-*α* [43], are involved in the development of SAP. It is critical for the therapy of SAP by reducing the cascade of cytokines at the early stages and ameliorating the disease and its systemic complications. The cerulein-induced pancreatitis was a sudden inflammation in the pancreas with extensive infiltration of leukocytes and excessive production of amylase and lipase. Picroside II treatment greatly reduced the infiltration of leukocytes and pancreas damage via the downregulation of IL-1*β*, IL-6, and TNF-*α* in SAP rats by inhibiting the expression of NF-*κ*B. NF-*κ*B is a key regulator of the expression of many inflammatory molecular. Inhibition of NF-*κ*B has been shown to improve survival rates in rats with taurocholate-induced pancreatitis [44]. Therefore, supplementation with picroside II could be an efficacious and promising remedy in the treatment for SAP.

Picroside II treatment increased the autophagic activities, which was showed by the expression or certain situation of LC3 (Figure 8(b)). However, the level of LC3 was still higher even the autophagic activities were very low (Figure 8(c)), suggesting that the autophagic activities might be associated with the situation of LC3 but not its level. Thus, we wanted to know how picroside II treatment could affect modified situation LC3 by exploring related molecules.

Picroside II treatment reduced the levels of NF-*κ*B, TNF-*α*, SIRT1, and LC3. The treatment could not affect the levels of TNF-*α*, SIRT1, and LC3 when NF-*κ*B was silenced, suggesting that picroside II treatment may affect the levels of TNF-*α*, SIRT1, and LC3 via NF-*κ*B. The overexpression of TNF-*α* is significantly correlated with the expression of NF-*κ*B [22], suggesting that the expression of TNF-*α* may increase the expression of NF-*κ*B or the expression of NF-*κ*B may improve the expression of TNF-*α*. To determine which one was determinant, NF-*κ*B was silenced and the results demonstrated NF-*κ*B silence resulted in the decrease of TNF-*α* level (Figure 8(c)). Meanwhile, the level of TNF-*α* was reduced and resulted in the decrease of SIRT1, and the increase of TNF-*α* resulted in the decrease of SIRT1, suggesting that the level of TNF-*α* was closely associated with the level of SIRT1. TNF-*α* may induce SIRT1 expression, which was accordant with the report of TNF-alpha-induced SIRT1 expression [23]. Theoretically, the increasing level of SIRT1 was correlated with the increasing level of LC3, which should increase the autophagic activities. The results did not demonstrate such a proposal. Thus, the acetylated situation of LC3 since SIRT1 was a kind of important deacetylases. The results showed that the level of acetylated LC3 was reduced when SIRT1 was increased, suggesting that the deacetylated LC3 should be increased since the total LC3 was increased. One thing should be paid here. In Figures 8, 9, and 10, only LC3 antibody was used but two forms LC3 I and LC3 II were detected. The C-terminal fragment of LC3 is cleaved to yield a cytosolic form as LC3 I. A subpopulation of LC3 I can be converted to an autophagosome-related form as LC3 II. Therefore, only one antibody can detect both forms of LC3.

**Figure 11:**
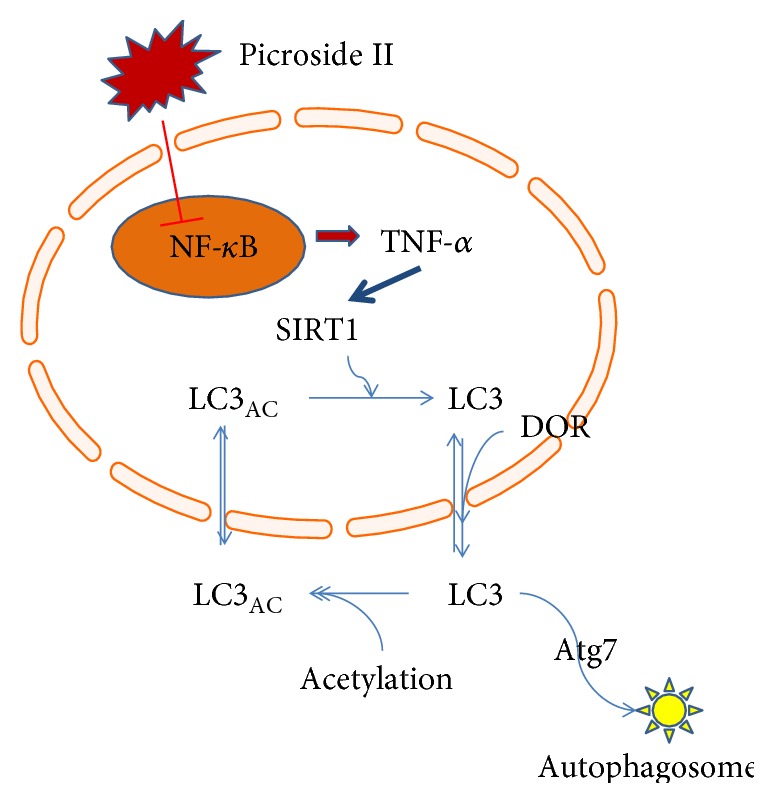
Schematic cartoon showing autophagic signaling pathways mediated by NF-*κ*B, TNF-*α*, SIRT1, and LC3 interactions.

Thus, present findings demonstrated that the overexpression of NF-*κ*B increased the expression of TNF-*α* and resulted in the increase of SIRT1. SIRT1 deacetylated LC3, which promoted autophagic activities as the main marker of autophagosome (Figure 11) [45]. Picroside II controlled the autophagic activity of pancreatic cells by affecting the expression of NF-*κ*B.

Certainly, there are some limitations for the present work. Although picroside II has been widely used for its protective functions, the exact mechanisms for its multiple functions are still unknown. Here, more functions of picroside II will be explored by using biochemical methodology and animal models. Present work is only limited to animals, and clinical trial is highly demanded. On the other hand, there is no signaling/mechanistic study for the antioxidant activity of picroside II against SAP (only SOD and GSH). How the antioxidant potential of picroside II mediate its action against SAP is not clear. According to an earlier report, antioxidants have opposite effects on the activation of NF-*κ*B [46]. Picroside II treatment can reduce the levels of NF-*κ*B, suggesting that the drug use may result in the increase of antioxidant activities. Therefore, to make sure the results, much work is still needed to be done in the future.

## 5. Conclusions

The present work found a potential molecular mechanism for the protecting functions of picroside II for SAP. Picroside II reduced the autophagic activity of SAP by inhibiting the expression of NF-*κ*B, TNF-*α*, and SIRT1 and reducing SIRT1-deacetylated LC3. Meanwhile, picroside II treatment showed anti-inflammatory activities by reducing the levels of IL-1*β*, TNF-*α*, and IL-6. Picroside II is promising by preventing the progression of SAP.

## Figures and Tables

**Table 1:** Histopathologic scoring system of SAP.

Diagnosis/score	Symptoms
Edema	
0	Absent
1	Focal expansion of interlobular septa
2	Diffuse expansion of interlobular septa
3	Diffuse expansion of interlobular septa, focal expansion of interacinar septa
4	Diffuse expansion of interlobular septa, diffuse expansion of interacinar septa
5	Diffuse expansion of interlobular septa, diffuse expansion of interacinar septa and increase in the distance between cells
Acinar cell necrosis	
0	Absent
1	1–4 necrotic cells
2	5–10 necrotic cells
3	11–16 necrotic cells
4	>16 necrotic cells
Hemorrhage	
0	Absent
1	1 area
2	2 areas
3	3 areas
4	More than 4 areas
Inflammation and perivascular infiltration	
0	0–1 interlobular or perivascular leukocyte
1	2–5 interlobular or perivascular leukocytes
2	6–11 interlobular or perivascular leukocytes
3	12–20 interlobular or perivascular leukocytes
4	>20 leukocytes or widespread microabscesses

**Table 2:** The histopathologic scores of SAP among different groups.

	Edema	Acinar cell necrosis	Hemorrhage	Inflammation and perivascular infiltration
Control	0^b,c,d,e^	0^b,c,d,e^	0^b,c,d,e^	0^b,c,d,e^
SAP model	3.5 ± 0.4^a,c,d,e^	2.9 ± 0.5^a,c,d,e^	3.0 ± 0.4^a,c,d,e^	4.1 ± 0.6^a,c,d,e^
Picroside II, 12.5 mg/kg	3.1 ± 0.3^a,b,d,e^	2.7 ± 0.3^a,b,d,e^	2.6 ± 0.3^a,b,d,e^	3.5 ± 0.4^a,b,d,e^
Picroside II, 25 mg/kg	2.5 ± 0.3^a,b,c^	2.2 ± 0.2^a,b,c^	2.3 ± 0.3^a,b,c^	3.0 ± 0.4^a,b,c^
Picroside II, 50 mg/kg	2.4 ± 0.2^a,b,c^	2.0 ± 0.2^a,b,c^	2.2 ± 0.2^a,b,c^	2.8 ± 0.3^a,b,c^

Note: control, healthy rats. SAP model, the rat model was induced with cerulean. Picroside II groups, SAP models were with different concentrations of picroside II. ^a^*P* < 0.05 versus the control group; ^b^*P* < 0.05 versus the SAP group; ^c^*P* < 0.05 versus the picroside II group (12.5 mg/kg); ^d^*P* < 0.05 versus the picroside II group (25 mg/kg); ^e^*P* < 0.05 versus the picroside II group (50 mg/kg).

**Table 3:** Biochemical parameters of enzyme activities for NASH.

Group (*n* = 10)	SOD (U/ML)	GSH (ng/L)	ALT	AST
Control	26.24 ± 3.36△●#■	25.14 ± 2.14△●#■	47.16 ± 10.48△●#■	105.32 ± 26.17△●#■
SAP model	14.25 ± 5.16∗#■	12.23 ± 1.83∗#■	88.78 ± 8.49∗#■	207.26 ± 17.64∗#■
Picroside II, 12.5 mg/kg	15.34 ± 2.32∗#■	13.28 ± 1.64∗#■	84.79 ± 12.36∗#■	201.46 ± 26.48∗#■
Picroside II, 25 mg/kg	21.34 ± 3.48∗△●	21.38 ± 1.02∗△●	69.45 ± 12.18∗△●	156.19 ± 27.28∗△●
Picroside II, 50 mg/kg	23.68 ± 2.44∗△●	22.28 ± 1.61∗△●	65.25 ± 15.8∗△●	153.24 ± 1744∗△●

Note: ^∗^*P* < 0.05 versus the control group (CG); ^△^*P* < 0.05 versus the model group (MG); ^●^*P* < 0.01 versus the picroside II group, 12.5 mg/kg; ^#^*P* < 0.05 versus the picroside II group, 25 mg/kg; ^■^*P* < 0.05 versus the picroside II group, 50 mg/kg.
